# Impulsivity and cannabis use disorder among tunisian sample

**DOI:** 10.1192/j.eurpsy.2023.1367

**Published:** 2023-07-19

**Authors:** B. Olfa, S. Rim, O. Reehab, A. Syrine, Z. Abdelmajid, C. Farah, M. Rim, F. Ines, M. Jawaher

**Affiliations:** 1 Psychiatry A Department, Hedi chaker university hospital; 2 Tunisian Association for the Fight against STDs and AIDS, sfax, Tunisia

## Abstract

**Introduction:**

In the past few years, there has been a considerable amount of evidence that cannabis use can cause structural and functional brain abnormalities.Structural imaging studies of cannabis users have revealed reduced prefrontal cortex volumes and white matter damage that may be involved with impulsivity. **Objectives:** To Determine the level of dependence on cannabis among cannabis users consulting the detoxification center of Sfax, Tunisia To assess in addition the impact of cannabis on impulsivity and motor control.

**Methods:**

This is a cross-sectional, descriptive and analytical study that was conducted over a period of 13 months between September 15, 2020 and October 1, 2021 among cannabis users consulting the detoxification center of Sfax, Tunisia.A short form of the Barratt Impulsiveness Scale (the BIS-15) and a Cannabis Abuse Screening Test (CAST) were used to assess impulsivity and to determine cannabis abuse. **Results:** Thirty Eight cannabis users agreed to participate in this study. The distribution of CAST scores showed that 36 users (94.7%) had problematic cannabis use at the time of the study. The mean BIS 15 score was 38.2. In our sample, The level of impulsivity was highest in people with a high level of cannabis dependence. A higher level of impulsivity was found in younger subjects. However, a greater level of impulsivity was found in subjects with a lower socio-economic level. Concerning employment status, unemployment was significantly correlated with a higher level of impulsivity.

**Conclusions:**

Impulsivity is often associated with a variety of problematic behaviors such as aggressive behavior, smoking, drug abuse, pathological gambling or compulsive buying.

A higher frequency of cannabis use and earlier age of onset use have been shown to be associated with the highest rates of impulsivity.

Therefore, cannabis addiction represents a real public health problem, both because of the serious complications and heavy repercussions that it causes.

**Disclosure of Interest:**

None Declared

